# Human echolocators adjust loudness and number of clicks for detection of reflectors at various azimuth angles

**DOI:** 10.1098/rspb.2017.2735

**Published:** 2018-02-28

**Authors:** L. Thaler, R. De Vos, D. Kish, M. Antoniou, C. Baker, M. Hornikx

**Affiliations:** 1 Department of Psychology, Durham University, Science Site, South Road, Durham DH1 3LE, UK; 2 Eindhoven University of Technology, 5600 MB Eindhoven, The Netherlands; 3 World Access for the Blind, Placentia 92870, CA, USA; 4 Department of Electronic Electrical and Systems Engineering, School of Engineering, University of Birmingham, Edgbaston, Birmingham B15 2TT, UK

**Keywords:** sonar, audition, blindness, beam-pattern, SNR

## Abstract

In bats it has been shown that they adjust their emissions to situational demands. Here we report similar findings for human echolocation. We asked eight blind expert echolocators to detect reflectors positioned at various azimuth angles. The same 17.5 cm diameter circular reflector placed at 100 cm distance at 0°, 45° or 90° with respect to straight ahead was detected with 100% accuracy, but performance dropped to approximately 80% when it was placed at 135° (i.e. somewhat behind) and to chance levels (50%) when placed at 180° (i.e. right behind). This can be explained based on poorer target ensonification owing to the beam pattern of human mouth clicks. Importantly, analyses of sound recordings show that echolocators increased loudness and numbers of clicks for reflectors at farther angles. Echolocators were able to reliably detect reflectors when level differences between echo and emission were as low as −27 dB, which is much lower than expected based on previous work. Increasing intensity and numbers of clicks improves signal-to-noise ratio and in this way compensates for weaker target reflections. Our results are, to our knowledge, the first to show that human echolocation experts adjust their emissions to improve sensory sampling. An implication from our findings is that human echolocators accumulate information from multiple samples.

## 1. Introduction

Echolocation is the ability to use reflected sound to infer spatial information about the environment. Just as in certain species of bats or marine mammals, people can echolocate by making their own sound emissions [1–4]. In fact, some people who are blind have trained themselves to use mouth clicks to echolocate. The beam pattern of mouth clicks that blind echolocators make exhibits a gradual 5 dB drop in intensity as function of angle from straight ahead to 90° to the side, but click energy is more heavily attenuated at further angles, and in particular at 135° sound energy drops by approximately 12 dB and at 180° (right behind the echolocator) by approximately 20 dB [5].

Detection of objects in echolocation depends on the echo-acoustic reflections they provide, and in bats it has been shown that echolocation behaviour is linked to the beam pattern of their emissions e.g. [6]. Since the beam pattern of human mouth clicks shows that click sound levels decrease at further azimuth angles it follows that the same reflector will be less effectively ensonified at further angles when compared to straight ahead. Therefore, based on the beam pattern of human mouth clicks we would predict that echolocation behaviour for object detection (i.e. to determine if an object is present or absent) should also change as a function of azimuth angle. Echolocating bats may shift spectro-temporal aspects of their calls (i.e. intensity, duration, spectrum, pulse rate) pending situational demands [7–12]. Bats may for example increase the intensity of their calls to compensate for a drop in echo intensity if targets are less effectively ensonified [13] and/or when ambient noise is present [14]. The possibility arises that human echolocators would also show adaptive emission behaviour if they are presented with reflectors that are less effectively ensonified, e.g. reflectors that are located off to the side when compared to in front of them. We might also expect a change in the accuracy of detection if targets are less effectively ensonified: [15] provided a model-based analysis estimating minimum level of reflected (echo) to direct (emission) sound (reflected-to-direct level difference, RDLD) that echolocators should be able to detect. Based on the analysis of a previous study [16] they suggested that the minimum RDLD for reflection delays between 4 and 15 ms should be between −22 and −19 dB. It would follow that people should not be able to detect reflectors with RDLDs less than −22 dB at a distance of 100 cm (delay approximately 6 ms). In the current study, we tested this hypothesis by calculating RDLDs based on acoustic measurements.

To date, there have not been any investigations of the dynamics of human echolocation behaviour, i.e. if people adjust their emissions to situational demands or not. Furthermore, ideas about minimum perceptible echo strength are based on acoustic models, but they have not been evaluated in people who have expertise in echolocation. Therefore, we here tested these ideas in a sample of eight blind expert echolocators. Specifically, the same 17.5 cm diameter circular disk was placed at 100 cm distance at 0°, 45° or 90°, 135° or 180° with respect to straight ahead. People's task was to use mouth-click based echolocation to determine if a reflector had been present or not. We recorded the acoustics of the task using microphones placed next to participants' ears. We analysed the recorded sound files to calculate acoustic properties of clicks and echoes.

We found that echolocators detected reflectors placed within the frontal hemisphere with 100% accuracy, but performance dropped to approximately 80% when the reflector was placed at 135° (i.e. somewhat behind) and to chance levels (50%) when placed right behind the echolocators (180°). Furthermore, echolocators increased loudness of clicks and also made more clicks for reflectors at angles 135°–180° when compared to reflectors at 0°–90°. There were no changes in spectral content, duration or inter-click intervals (ICIs).

Level differences in terms of overall sound energy between echo and emission (i.e. RDLD [15]) ranged from −11 dB (0°), −14 dB (45°), −18 dB (90°), −27 dB (135°) and −31 dB (180°). This implies that expert echolocators failed to perceive RDLDs of −31 dB (180°), but that they were able to reliably detect RDLDs as low as −27 dB (135°) in our study (i.e. at onset delays of approximately 6 ms). Measuring echo intensity revealed that changes in echo strength as function of angle follow the same pattern as changes in RDLD, but that echo strength drops less than RDLD. This can be explained by the fact that increases in click intensity as function of angle will ‘boost’ echo intensity, i.e. making clicks louder will also make echoes louder. Yet, because RDLD is computed as the difference between echo and click, and this difference remains even if both click and echo become louder, RDLDs are left unchanged by the boost in click intensity.

Close temporal proximity of clicks and echoes in our study (onset delay approximately 6 ms) implies that detection of echoes takes place within a temporal window for which forward masking (of the echo by the emission) which sometimes goes into simultaneous masking (when click duration exceeds echo delay) [17,18] and/or echo suppression [19,20] are relevant. Even though research suggests that echo suppression is reduced in active echolocation, it is nonetheless present and affects performance [21]. The reason that an increase in click intensity (as well as numbers of clicks) is a useful strategy to increase detection performance, is because of the nonlinear behaviour of masking [17,18].

In the following sections we describe the methods and results, before discussing the implications of our findings.

## 2. Material and methods

### (a) Participants

Eight blind participants with experience in echolocation took part in the experiment. Participant details are listed in table 1. All participants (except S1) had normal hearing as assessed with pure tone audiometry (500–8000 Hz). S1 had hearing loss (approx. 15 dB) from 500–4000 Hz.

**Table 1.** Details of participants who took part in the study.

participant ID	gender	age at time of testing	cause of vision impairment	severity of vision impairment at time of testing	age at onset of vision impairment	age at start of using mouth-click based echolocation
S1	male	53	optic nerve compression	right eye total blindness; left eye bright light detection (tested with blindfold)	5 years	43 years
S2	female	41	Leber's congenital amaurosis	total blindness	birth	31 years
S3	male	49	retinoblastoma	total blindness	birth; enucleation at 1 year	<3 years
S4	male	33	optic nerve atrophy	total blindness	14 years	15 years
S5	male	56	retinal detachment	bright light detection (tested with blindfold)	birth	6 years
S6	male	43	Leber's congenital amaurosis	bright light detection right eye; total blindness left eye; (tested with blindfold)	birth	33 years
S7	male	34	glaucoma	total blindness	gradual loss since birth	12 years
S8	male	32	optic nerve atrophy	bright light detection (tested with blindfold)	8 years	29 years

### (b) Setup and apparatus

**Figure 1.**
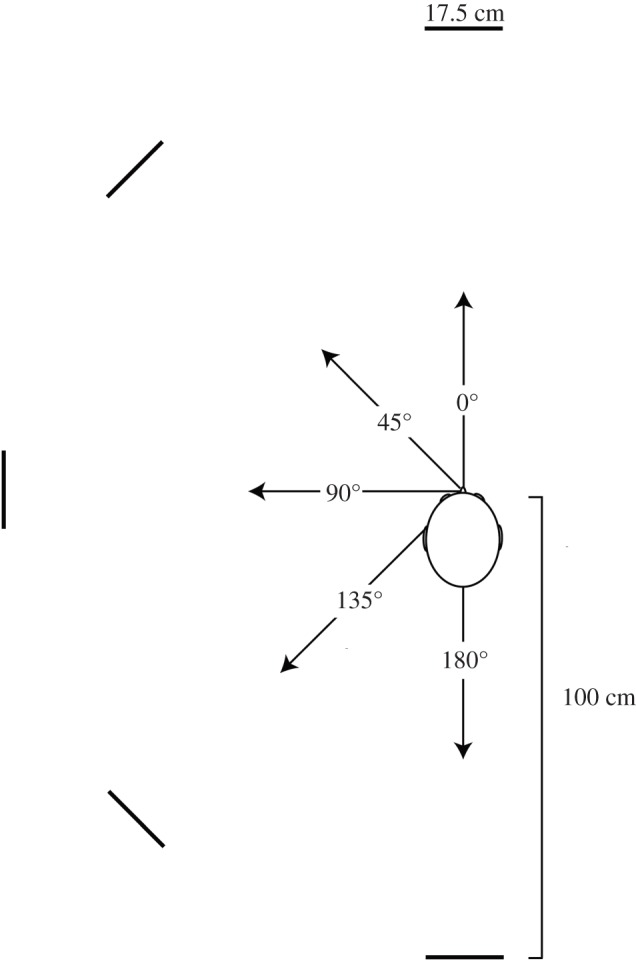
Sketch of the experimental setup as seen from above. The reflector was a 17.5 cm diameter circular disc made from 5 mm thick wood. The reflector always faced the participant and was presented at 100 cm distance. Each location was tested separately, but we have drawn reflectors at each location for illustration of reflector orientation with respect to the participant. Relative dimensions drawn approximately, not to scale.

All testing was conducted in a 2.9 m × 4.2 m × 4.9 m noise insulated and echo dampened room (walls and ceiling lined with foam wedges with cut-off frequency 315 Hz; floor covered with foam baffles, noise floor 24dBA). Participants stood in the centre of the room. Tactile markers were used to allow participants to reliably place their head at the same position throughout a trial, while not impeding movements of the mouth for clicking. The reflector was a 17.5 cm diameter 5 mm thickness wooden disk, presented at mouth level at 100 cm distance on top of a 0.5 cm diameter steel pole (17.5 diameter comprises 10° acoustic angle at 100 cm). A reflector could be presented at 0°, 45°, 90°, 135° and 180° to the left of the participant. The reflector always faced the participant. Figure 1 illustrates the setup. We made recordings of testing sessions with microphones placed on either side of the participant's head, next to the tragus of each ear (DPA SMK-SC4060 miniature microphones; DPA microphones, Denmark; TASCAM DR100-MKII recorder; TEAC Corporation, Japan; 24 bit and 96 kHz).

### (c) Task and procedure

Participants placed their head in the centre of the room facing straight ahead. The head had to be kept straight ahead for the duration of a trial. A reflector could be presented at 0°, 45°, 90°, 135° and 180° to the left of the participant always at 100 cm distance. The participant's task on every trial was to make mouth clicks and to judge vocally if there was a reflector present or not. Participants received feedback (correct or incorrect response). Reflectors were present on 50% of the trials, and absent otherwise. The order in which locations were tested was as follows. The first 10 trials were presented at 0°, followed by 10 trials at 45°, then 135°, etc. up to 180°. This was followed by a break. Then testing was resumed starting at 180° going to 0°. A total of 20 trials were done for each location. Within each location, the order of present and absent trials was randomized. For each location participants were made familiar with the task, and given the opportunity of two practice trials. We instructed participants to give a response whenever they felt they were ready to do so (i.e. there was no limit on trial duration). We instructed them to go with their ‘best guess’ if they felt unable to reach a decision otherwise. Total testing time was approximately 45 min for each participant. Participants were asked to block their ears and hum in between trials. The start of a trial was indicated to the participant via a tap on their foot (using a long cane). The participant then unblocked their ears and commenced the trial.

### (d) Data analysis

#### (i) Behaviour and acoustics

To characterize detection performance we computed percentage correct detections for each location.

To characterize participants clicking behaviour we analysed recorded sound files for each participant. Analysis were done using Matlab (The Mathworks, Natick, USA). We analysed the numbers of clicks made for each trial, duration, intensity, ICIs and click power spectra, as well as peak frequency, power spectral centroid and bandwidth based on power spectra. We also computed the level difference between reflected sound (echo) and direct sound (click) (RDLD), and echo intensity (dB SPL). This was done to characterize participant's echo-acoustic sensitivity. The number of clicks for each trial was determined visually and acoustically by visual and acoustic screening of the sound files. During this process, clicks were also isolated from intermittent speech and other background noise for further analysis. Click duration was computed as the time from click onset to offset. To obtain onset and offset we first computed the click envelope as the absolute value of signal and smoothing it with a 40 sample (0.42 ms) moving average. Click onset was determined as the first point where envelope value exceeded 5% (−26 dB) of the maximum. The offset was determined by fitting a decaying exponential to the envelope (starting from envelope maximum; performing a nonlinear least-squares fit with a trust-region algorithm implemented in the Matlab optimization toolbox) and determining where the fitted curve dropped to 5% (−26 dB) of maximum. Click intensity was computed as root mean square (RMS) intensity of clicks for the duration of the click. To characterize spectral content of clicks we computed each click's power spectrum and then determined the peak frequency, power spectral centroid and bandwidth (using a 25 dB drop relative to peak [22], and using the powerbw.m function implemented in the Matlab signal processing toolbox) for each trial, and then averaged across trials for each location. We also calculated the (amplitude) spectral centroid, as well as bandwidth based on a 3 dB and based on a 10 dB drop (results provided in the electronic supplementary material, Results S1). To compute RDLD, which only applies to reflector present trials, we determined click and echo RMS intensity, and then took the difference. The echo was detected by windowing of the sound at the expected time of the echo (because the reflector had been placed at 100 cm distance), and determining on- and offset using the same method as used for clicks. We imposed the additional criterion that echo duration could not exceed click duration. For two participants RDLDs could not be computed because these participant's click durations exceeded echo onset time. Since duration estimates will affect RMS calculations, we also calculated click intensity and RDLDs based on peak intensity values that are not affected by duration estimates (results provided in the electronic supplementary material, Results S1).

#### (ii) Statistical analysis

To investigate effects of reflector location (0°, 45°, 90°, 135° and 90°) on detection and clicking behaviour we subjected data to repeated-measures ANOVA. Pairwise comparisons were done using *t*-tests (paired samples). For all analyses statistical significance was determined using an *α* level of 0.05. Greenhouse Geisser correction was applied if the sphericity assumption could not be upheld.

## 3. Results

**Figure 2.**
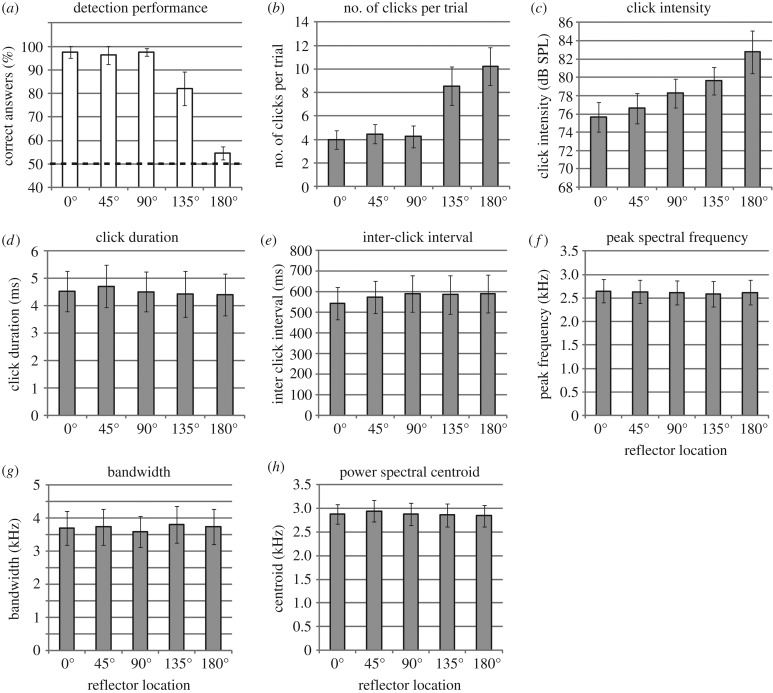
Measures of echolocation behaviour. Bars are means and errors bars standard error of the mean (s.e.m.) across people. People's detection performance (*a*), numbers of clicks per trial (*b*) and click intensity (*c*) change across testing locations, but click duration (*d*), ICI (*e*), click peak frequency (*f*), click bandwidth (25 dB drop) (*g*) and click power spectral centroid (*h*) remain unchanged.

People's detection performance is shown in figure 2(*a*). It appears that performance is stable across reflector locations 0°, 45° and 90°, but drops for 135° and 180°. Consistent with this the main effect of location was significant (*F*_1.628,11.396_ = 33.767; *p* < 0.001; 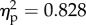), and linear (*F*_1,7_ = 152.482; *p* < 0.001; 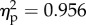) and quadratic trends (*F*_1,7_ = 56.952; *p* < 0.001; 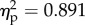) were significant as well. Follow up *t*-tests showed that while performance did not decrease from 0° to 45° (*p* = 0.351) and from 45° to 90° (*p* = 0.685), it decreased significantly from 90° to 135° (*p* = 0.043), and from 135° to 180° (*p* = 0.006). One sample *t*-tests showed that performance was significantly better than chance in locations 0° (*t*_7_ = 19.0; *p* < 0.001), 45° (*t*_7_ = 12.333; *p* < 0.001), 90° (*t*_7_ = 29.023; *p* < 0.001) and 135° (*t*_7_ = 4.472; *p* = 0.003), but that it did not differ from chance at 180° (*t*_7_ = 1.62; *p* = 0.149).

**Figure 3.**
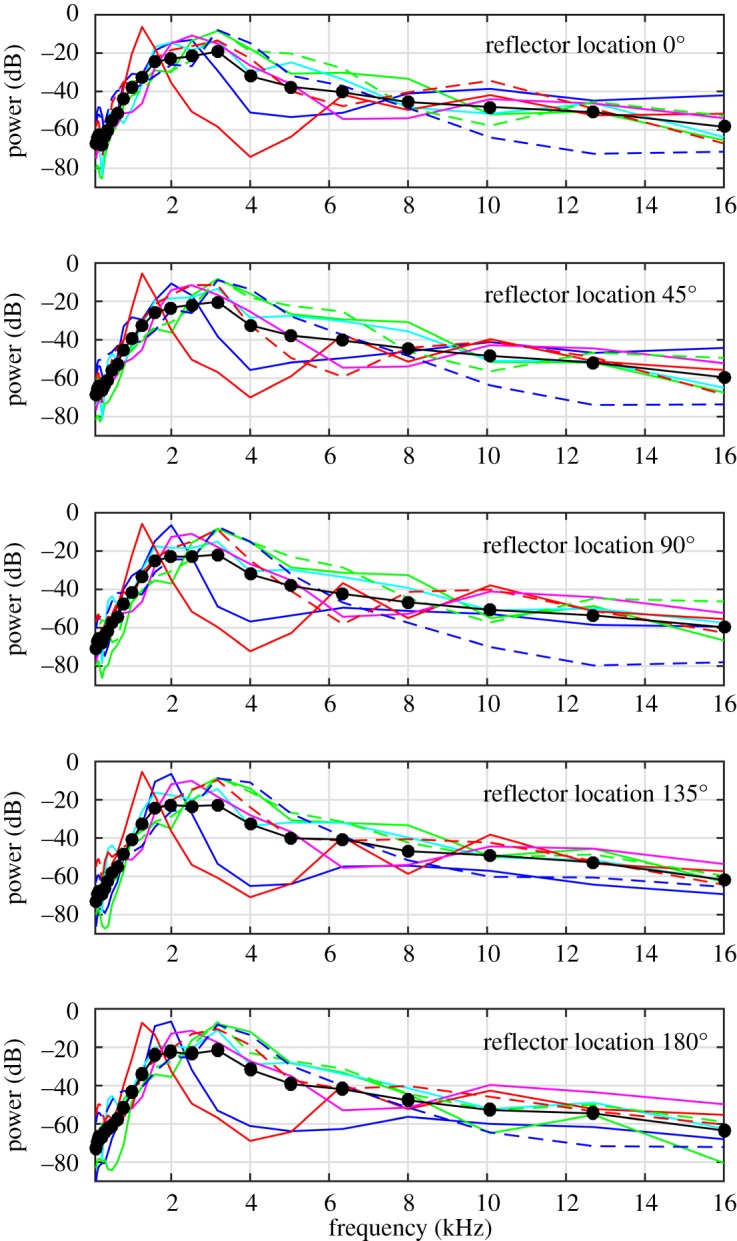
Power spectra (1/3 octave bands with respect to total power) for the different testing locations. Thin lines denote data for individual participants, where the same line colours and types denote data from the same participant across testing locations. Thick lines and symbols denote the average across participants. Spectral content of clicks remains unchanged across testing locations.

Focusing on people's clicking behaviour, it is evident that for farther angles people increased the number of clicks they made (figure 2(*b*)) and the intensity of their clicks ((*c*)). With respect to the numbers it appears that people make the same numbers of clicks per trial across locations 0°, 45° and 90°, but that they increase numbers for locations 135° and 180°. Consistent with this the main effect of location was significant (*F*_1.830,12.811_ = 14.967; *p* = 0.001; 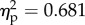), and linear (*F*_1,7_ = 22.134; *p* = 0.002; 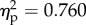) and quadratic trends were significant as well (*F*_1,7_ = 10.929; *p* = 0.013; 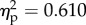). The fourth-order trend was also significant (*F*_1,7_ = 10.112; *p* = 0.015; 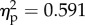). Follow up *t*-tests showed that while numbers of clicks did not increase from 0° to 45° (*p* = 0.266) and from 45° to 90° (*p* = 0.498), they increased significantly from 90° to 135° (*p* = 0.005), but then again remained the same from 135° to 180° (*p* = 0.227). With respect to click intensity it appears that people steadily increase the intensity of their clicks as angles become more eccentric. Consistent with this the main effect of location was significant (*F*_1.377, 9.640_ = 4.931; *p* = 0.043; 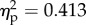), and the linear trend was significant as well (*F*_1,7_ = 6.352; *p* = 0.040; 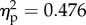). Follow up *t*-tests showed that while click intensity did not increase from 0° to 45° (*p* = 0.184) and from 45° to 90° (*p* = 0.165), it increased significantly from 90° to 135° (*p* = 0.031), but then again did not differ significantly from 135° to 180° (*p* = 0.143). The same pattern of results was obtained based on peak intensity values (electronic supplementary material, Results S1). Click duration, ICIs, click peak frequency, bandwidth and power spectral centroid remained stable across testing locations (figure 2(*d*)–(*h*)), and consequently none of the ANOVAs revealed significant effects of location for these measures. The same pattern of results was obtained for the (amplitude) spectral centroid and for bandwidth using drop values of 3 and 10 dB (electronic supplementary material, Results S1). The fact that spectral content did not change is also evident in figure 3, which shows that power spectra (1/3 Octave Bands) did not change across testing locations.

**Figure 4.**
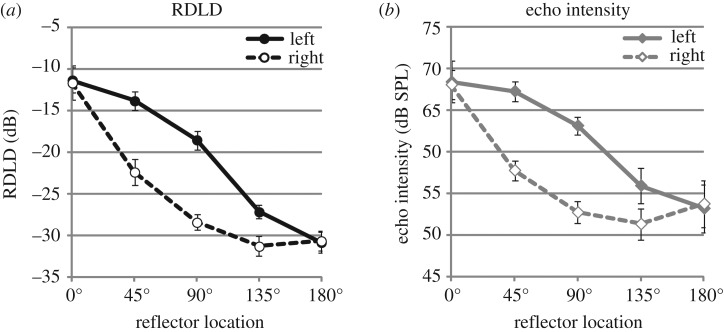
RDLDs (*a*) and echo intensity (*b*) for right and left channels separately. Symbols are means and errors bars s.e.m. across people. RDLDs and echo intensity decrease at further angles.

To characterize the acoustics further we calculated RDLDs for right and left channels separately. Data are shown in figure 4(*a*). Echo intensities (i.e. only intensity of the reflected sound) are shown in figure 4(*b*). With respect to RDLDs it is evident that they decrease as reflectors are located at further testing angles. It is also evident that RDLDs are generally higher for the left when compared to the right channel, except for 0° and 180° testing locations. The decrease of RDLDs at further testing angles was expected because the beam pattern of mouth clicks causes reflectors at further angles to be less well ensonified, thus returning weaker echoes. On the other hand, because the relative positioning of mouth to ear is fixed, the click as heard through each channel remains the same regardless of testing location. As a result, the relative strength of the reflected sound (echo) when compared to the direct sound (click), which is measured in RDLDs, decreases at further angles. The effect that RDLDs are generally higher for the left when compared to the right channel, except for 0° and 180° testing locations was also expected because reflectors at 45°, 90° and 135° testing locations were presented on the left side, thus leading to attenuation of reflected sound for the right when compared to the left channel for those locations. Consistent with these expectations the ANOVA revealed a significant effect of location on RDLD (*F*_4,20_ = 68.422; *p* < 0.001; 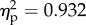), a significant effect of ‘channel’ (*F*_1,5_ = 21.947; *p* = 0.005; 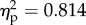), and a significant location × channel interaction (*F*_4,20_ = 12.045; *p* < 0.001; 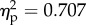). Follow up *t*-tests showed that RDLDs differed significantly between left and right channels at 45° (*t*_5_ = 5.078; *p* = 0.004), 90° (*t*_5_ = 5.575; *p* = 0.003) and 135° (*t*_5_ = 2.660; *p* = 0.045), but not at 0° (*t*_5_ = 0.188; *p* = 0.858) or 180° (*t*_5_ = 0.304; *p* = 0.773). The same pattern of results was obtained based on peak intensity values (electronic supplementary material, Results S1).

With respect to echo intensity (figure 4(*b*)) it is evident that they follow the same pattern as RDLDs, but that the decrease in echo intensity going from straight ahead to further angles is less than decrease in RDLD. For example, while RDLDs drop approximately 19 dB from 0° to 180° the corresponding drop in echo intensity is only approximately 14 dB. This can be explained by the fact that for further angles participants increase the intensity of their clicks (approx. 7 dB from 0° to 180°). A boost in click intensity will also boost echo intensity, but will leave RDLDs unaffected because RDLDs depend on both click intensity and echo intensity.

## 4. Discussion

Our results clearly demonstrate that people, just like bats, adjust their emissions to situational demands. In our study, people adjusted the intensity and number of clicks they made. Increasing the intensity of clicks leads to an increase in echo intensity. Therefore, it is likely that people (just like bats [13,14]) increased click intensity to increase signal-to-noise ratio (SNR), where the signal is the echo and noise is residual ambient noise and/or noise intrinsic to the human auditory system. Close temporal proximity of clicks and echoes in our study (onset delay approximately 6 ms) implies that detection of echoes will be affected by forward masking (of the echo by the emission) which sometimes goes into simultaneous masking (when click duration exceeds echo delay) [17,18] and/or echo suppression [19,20]. The reason that an increase in click intensity is nonetheless a useful strategy to increase detection performance (by increasing SNR) is because of the nonlinear behaviour of masking [17,18]. Increasing the number of clicks is expected to have the same purpose, i.e. to increase SNR. In fact, artificial systems and applications make use of this by averaging across multiple samples in order to increase SNR. An important implication from this is that human echolocators must accumulate information from multiple samples over time. We did not find evidence for changes in spectral content, click duration or ICIs. This does not rule out that these aspects might change in other contexts, however.

Recordings in our study were made next to the tragus of each ear. Nonetheless, even though our measurements do not allow us to describe intensity of the click signal as measured at the mouth, our measurements are well suited to quantify changes in transmitted click intensity across conditions. Specifically, even though changes in sound intensity measured at the ear can be owing to changes either in intensity of the sound made at the mouth or changes in directionality of the sound, directionality of sounds can only be altered by changing the shape of the mouth, i.e. increasing mouth aperture. Importantly, however, changes in mouth aperture that would lead to changes in intensity as measured at the ear in our current study (e.g. approximately 7 dB from 0° to 180°) would also cause substantial changes in spectral content of the clicks, because changes in the aperture of the human mouth affect both directionality and spectral content [23,24]. In our study, we did not observe any change in spectral content across conditions. As a consequence, changes in click intensity that we measured at the ear must be owing to changes in intensity of the clicks, rather than changes in directionality.

In bats, adaptive behaviour has been observed as well. For example, some species may shift spectro-temporal aspects of their calls (i.e. intensity, duration, spectrum, pulse rate) pending on the environmental conditions [7–14], or they may adjust the direction and/or width of their sound beam when they lock onto a target [6,7,25,26]. Humans can of course adjust click direction by moving their head. Since head movements were not permitted in our study, we did not measure dynamic adjustments in terms of head rotation. Nonetheless, it has been shown that human echolocation can be facilitated by head movement [27–29]. Based on our current results, we suggest that future work should characterize these movements with respect to echo-acoustic sampling. The paradigm we used here did not require self movement of the echolocators, or approach of a target, and it is possible that for this reason we did not observe changes in ICI, click duration or spectrum, that are typically observed in bats during target approach. Nonetheless, the changes in behaviour (and RDLD) that we observed in our study are consistent with changes that one might expect based on the transmission characteristics of mouth clicks that expert echolocators make [5,30], and we also show that human echolocation behaviour is a dynamic process. This raises the possibility that human echolocation may be governed by similar principles as echolocation in bats.

Participants in our study performed better than chance for 0°, 45°, 90° and 135°, but not at 180°. This implies that despite increased echo intensity and multiple samples the echo signal was not reliable enough to support accurate performance at 180°. At 180° the difference between reflected and direct sound (i.e. RDLD) in our study was −31 dB and echo intensity was 53 dB SPL. While for normal hearing sound levels of 53 dB SPL are readily audible, the likely reason that an echo of this magnitude did not support reliable performance in our participants was that they followed the much louder click in brief succession (echoes were 31 dB softer than clicks, i.e. less than 2.8% intensity). As mentioned above, echo perception in our study took place within a temporal window for which forward masking (of the echo by the emission) which sometimes goes into simultaneous masking (when click duration exceeds echo delay) [17,18] and/or echo suppression [19,20] are relevant for human hearing. Even though research suggests that echo suppression is reduced in echolocation, it is nonetheless present and affects performance [21]. Thus forward (or simultaneous) masking and/or echo suppression are the likely explanation for why echolocators did not detect echoes at sound levels of 53 dB SPL in 180° conditions. At the same time, RDLD for 135° was −27 dB in the left channel (and −31 dB in the right channel), and echo intensity was 56 dB SPL (left) and 51 dB SPL (right). Since performance for 135° with approximately 80% was better than chance this implies that our participants could successfully perform when RDLD was as low as −27 dB and the echo was 56 dB SPL. This suggests that under these conditions effects of forward masking and/or echo suppression could be overcome by our participants. Another possibility is that in these conditions participants were able to rely on a binaural intensity cue to perform the task [31]. Such binaural cues were absent at 180° (compare figure 3). It has been shown that echolocating bats (big brown bats) can detect echoes at RDLDs as low as −90 dB at a target distance of 80 cm (delay of 4.8 ms) [32]. The measurement setup in [32] was slightly different in that intensity of the emission (direct sound) was measured 10 cm in front of the bat's mouth and the intensity of the echo was measured as it was delivered to the bats ear. Nonetheless, RDLDs measured for bats would still be well below the values we have shown here for people.

Previous work done by [15] had estimated ‘best’ RDLDs for human echolocators to be between −22 and −19 dB for echo delays between 4 and 15 ms. These estimates were based on acoustic modelling using a previously published study to estimate RDLDs and audibility thresholds [16]. RDLD values of −19 to −22 were already well below those for human audibility thresholds for single reflections based on external signals (e.g. noise bursts), which are more around −15 dB for delays between 5 and 7 ms [33,34]. Our results based on analyses of RDLDs clearly demonstrate that echo-acoustic sensitivity in our sample of eight echolocation experts is much better than expected based on previous estimates. This emphasizes the adaptation of the human auditory system in human echolocation experts. It also highlights that in order to understand how human echolocation works there is a need to conduct behavioural work in human echolocation experts in addition to acoustic modelling.

The results reported here were obtained with a circular disk reflector of 17.5 cm diameter. Reflector size was kept unchanged because the variable under investigation was reflector location. Based on our analyses of echo intensity and RDLDs we would predict, however, that increasing reflector size would enable reliable performance even at 180°, i.e. behind the echolocators at 100 cm, as long as RDLDs of −27 dB or better and echo intensity of 56 dB SPL or better can be achieved. This is because these are the lowest values that were reliably detected in our study (i.e. at 135°).

In the current study, sound measurements made next to the tragus of each ear, while in [5] recordings of clicks were made within the horizontal/vertical planes. Nonetheless, the spectro-temporal pattern of clicks that we measured here were similar to those reported in [5], with the exception that two participants in our current study made clicks of longer duration.

In our study, participants were not permitted to move their head because the goal was to measure changes in emission and detectability as function of angle. It was evident from discussing the task with each participant, however, that they would typically use head movements to get better impressions of objects located at farther angles. Nonetheless, in everyday situations it is often not known in advance where an object might be. Therefore, detection of objects at farther angles is required also during regular echolocation processes.

In conclusion, our results are, to our knowledge, the first to demonstrate that human echolocators adjust their sound emission strategies to improve sensory sampling, highlighting the dynamic nature of the echolocation process in humans.

## Supplementary Material

Supplemental Results S1

## Data accessibility

This article has no additional data.
