# Supplementary material for: Human echolocators adjust loudness and number of clicks for detection of reflectors at various azimuth angles
Source: Proc Biol Sci. 2018 Feb 28;285(1873):20172735. doi: 10.1098/rspb.2017.2735 (PMC5832709; doi:10.1098/rspb.2017.2735)
Supplement: Supplemental Results S1 [file rspb20172735supp1.pdf]

Human Echolocators adjust loudness and number of clicks for detection of reflectors at various azimuth angles

Thaler, L., De Vos, R., Kish, D., Antoniou, M., Baker, C., Hornikx, M.

Supplemental Results S1

Results for Bandwidth based on 3dB drop w/r to peak (instead of 25dB)

Data are shown in Figure S1. Consistent with results from bandwidth values based on a 25dB drop w/r to peak there is no change of bandwidth (3dB drop) across locations.

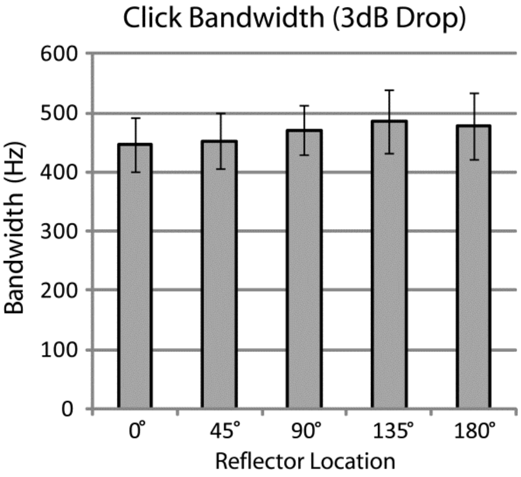

Figure S1 – Bandwidth (based on 3dB drop) across testing locations. Bars are means and errors bars standard error of the mean (SEM) across people. There is no change in bandwidth across locations.

Results for Bandwidth based on 10dB drop w/r to peak (instead of 25dB)

Data are shown in Figure S2. Consistent with results from bandwidth values based on a 25dB drop w/r to peak there is no change of bandwidth (10dB) across locations.

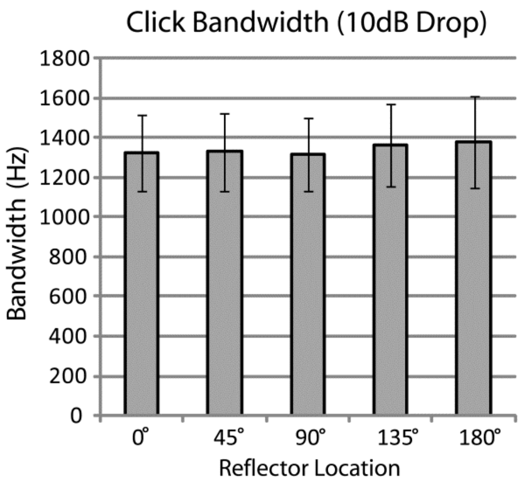

Figure S2 – Bandwidth (based on 10dB drop) across testing locations. Bars are means and errors bars standard error of the mean (SEM) across people. There is no change in bandwidth across locations.

Results for Spectral Centroid (instead of Power Spectral Centroid)

Data are shown in Figure S3. Consistent with results from power spectral centroid, there is no change in spectral centroid (amplitude based) across locations.

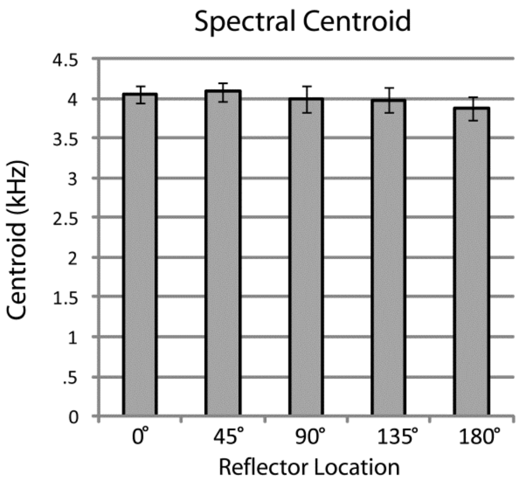

Figure S3 – Spectral Centroid (Amplitude based) across testing locations. Bars are means and errors bars standard error of the mean (SEM) across people. There is no change in spectral centroid across locations.

Results for click intensity based on peak intensity (instead of RMS)

Data are shown in Figure S4. Consistent with results from intensity values based on RMS, people steadily increase the peak intensity of their clicks as angles become more eccentric. Consistent with this the main effect of location was significant ($F(1.349, 9.445)=5.044$; $p=.042$; $\eta^2_p = .419$), and the linear trend was significant as well ($F(1,7)=6.357$; $p=.040$; $\eta^2_p = .476$). Follow up t-tests showed that whilst click peak intensity did not increase from 0° to 45° (p=.184) and from 45° to 90° (p=.156), it increased significantly from 90° to 135° (p=.036), but then again did not differ significantly from 135° to 180° (p=.150).

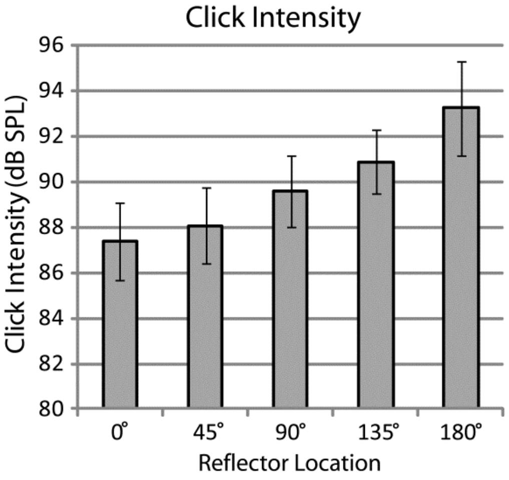

Figure S4 – Click intensity (based on peak values) across testing locations. Bars are means and errors bars standard error of the mean (SEM) across people. Intensity increases at further angles.

Results for RDLDs based on peak intensity (instead of RMS)

Data are shown in Figure S5. Consistent with results from RDLD values based on RMS, RDLDs decrease as reflectors are located at further testing angles. It is also evident that RDLDs are generally higher for the left as compared to the right channel, except for 0° and 180° testing locations. Consistent with results for RDLD values based on RMS, ANOVA revealed a significant effect of location on RDLD ($F(4,20)=86.794$; $p<.001$; $\eta^2_p = .946$), a significant effect of 'channel'

(F(1,5)=27.515; p=.003; η^2_p = .846), and a significant location x channel interaction (F(4,20)=11.927; p<.001; η^2_p = .705). Follow up t-tests showed that RDLDs differed significantly between left and right channels at 45° (t(5)=4.391; p=.007), 90° (t(5)=6.491; p=.001) and 135° (t(5)=3.492; p=.017), but not at 0° (t(5)=.061;p=.954) or 180° (t(5)=.014; p=.989).

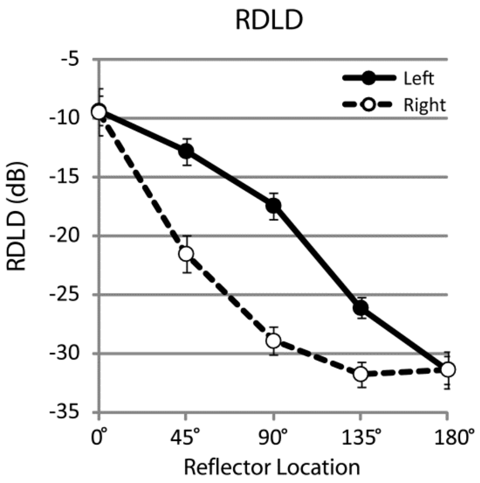

Figure S5 - RDLDs (based on peak intensity values) for right and left channels separately. Symbols are means and errors bars SEM across people. RDLDs decrease at further angles.
